# Supplementary material for: Correction: Real-time detection of microgrid islanding considering sources of uncertainty using type-2 fuzzy logic and PSO algorithm
Source: PLoS One. 2021 Nov 9;16(11):e0259925. doi: 10.1371/journal.pone.0259925 (PMC8577727; doi:10.1371/journal.pone.0259925)
Supplement: S1 File — (PDF) [file pone.0259925.s001.pdf]

RESEARCH ARTICLE

Real-time detection of microgrid islanding considering sources of uncertainty using type-2 fuzzy logic and PSO algorithm

Taghi Hosseinzadeh Khonakdari[1], Mehrdad Ahmadi Kamarposhti[2]*

1 Department of Electrical Engineering, Islamic Azad University, Sari, Iran, **2** Department of Electrical Engineering, Islamic Azad University, Jouybar, Iran

* m.ahmadi@jouybariau.ac.ir, mehrdad.ahmadi.k@gmail.com

Abstract

In this paper, a new innovative type-2 fuzzy-based for microgrid (MG) islanding detection is proposed in the condition of uncertainties. Load and generation uncertainties are two main sources of uncertainties in microgrids (MGs). Regardless of the uncertainties, the results cannot be confirmed. The proposed controller detects islanding in the fastest time under different conditions, and the uncertainties in the system will not considerably affect the controller's performance. The proposed method is simulated on the sample system and examined in different scenarios. Then, a comparison is made in different conditions and scenarios between the suggested method and some common methods that have been presented so far to demonstrate the ability of the proposed method for islanding detection.

Check for updates

OPEN ACCESS

Citation: Hosseinzadeh Khonakdari T, Ahmadi Kamarposhti M (2021) Real-time detection of microgrid islanding considering sources of uncertainty using type-2 fuzzy logic and PSO algorithm. PLoS ONE 16(9): e0257830. https://doi.org/10.1371/journal.pone.0257830

Editor: Usman Qamar, National University of Sciences and Technology (NUST), PAKISTAN

Received: March 12, 2021

Accepted: September 12, 2021

Published: September 28, 2021

Copyright: © 2021 Hosseinzadeh Khonakdari, Kamarposhti. This is an open access article distributed under the terms of the Creative Commons Attribution License, which permits unrestricted use, distribution, and reproduction in any medium, provided the original author and source are credited.

Data Availability Statement: All relevant data is within the paper.

Funding: The authors received no specific funding for this work.

Competing interests: The authors have declared that no competing interests exist.

1. Introduction

The MG islanding must be identified as soon as possible and appropriate control orders issued. In the case the predetermined MG islanding, it is easier to control the voltage and frequency beforehand. But, if the islanding occurs unintentionally and during the connection of a heavy load, it will cause instability. In the case of islanding, the voltage and frequency changes of the islanded section are slow and the protective devices will not be able to detect the formation of the island [1].

Identifying the islanding status is an important issue in MGs connection, which has been the subject of research in recent years. Necessary conditions for becoming an island in microgrids have been published in a number of standards, such as IEEE-1574 and IEC-62116 [2]. The methods of identifying islanding are generally divided into two categories: "remote" and "local" detection methods [3, 4]. Remote methods are based on telecommunication systems between the operator and distributed generation (DG), while local methods use information collected at DG locations. Remote methods have a fast response time, lack of non-detection zone (NDZ), and they are highly reliable. Nonetheless, the disadvantage of this method is the relatively high cost of their implementation and maintenance.

Local detection methods can be categorized into passive, active, hybrid, and intelligent methods, which operate by measuring SG parameters, such as voltage, current, frequency, and

harmonic distortion on the microgrid side [5]. Passive methods include under/overvoltage relays (UVR/OVR), under/over-frequency relays (UFR/OFR) [5], rate of change of frequency (ROCOF) [6], methods based on rate of change of active power (ROCOAP) and rate of change of reactive power (ROCORP) [7], a technique based on two criteria of transient index value (TIV) and positive sequence of current angle at common connection point [8], detection methods using signal processing techniques [9], methods based on unbalance voltage (UV) [2], methods based on total harmonic distortion (THD) [10], and methods based on differential transient rate of change of frequency (DTROCOF) [11].

Active methods operate on the basis of a disruption and collecting its effects, and include a sudden increase in THD [12], Sandia frequency shift (SFS) [13], Sandia voltage shift (SVS) [14], active frequency deviation (AFD) [15], current injection [16], negative-sequence current injection [17], negative-sequence voltage injection [18], high frequency signal injection [19], traveling wave theory [20], virtual capacitor [21], dual second-order generalized integrator-phase locked loop (DSOGI-PLL) [22], voltage phase angle [23], current injection and voltage monitoring [24], and positive frequency based on frequency locked loop (FLL) [25].

In hybrid methods, the capabilities of active and passive methods have been used to detect islanding faults. Ref. [26] presents a hybrid method based on the Gibbs phenomenon for identifying islands based on a combination of ROCOF methods at a given moment and measuring the THD. The hybrid method based on active and passive algorithms that use the voltage phase angle (VPA) and the voltage unbalance (VU) is presented in [27, 28]. Authors in [29] utilize a network impedance estimation method that uses resonant excitation when a fault occurs in the network. In [30], the hybrid method of islanding detection and priority-based load curtailment has been used in distribution networks in the presence of DG units. In [31], a hybrid islanding detection system is introduced based on an inverter that acts as a virtual synchronous generator.

Over time, with the growth of intelligent methods, attention has been focused on using these methods to identify islanding cases. Examples of such methods include the decision-making tree (DT) [32], support vector machine (SVM) [33], artificial neural network (ANN) [34], fuzzy logic control (FLC) [35], and adaptive neuro-fuzzy inference system (ANFIS) [36], which are used to categorize different conditions. In [37], graph search method is employed to determine the islanding operation of renewable energy sources RESs and the main grid based on system configuration. A new method of islanding detection is proposed in [38] for photovoltaic systems (PVs) connected to the main grid using the maximum power point (MPP) tracking algorithm. In [39], morphological filters along with experimental modal analysis (EMD) have been used to implement islanding adaptive signal detection.

The previously introduced intelligent methods for islanding mode identification suffer from two important drawbacks: inability to identify the islanding mode in the short term and disregarding uncertainty in the power system. Several me intelligent methods are unable to detect the islanding mode in a short time due to the complex logic behind them. On the other hand, the methods that satisfy the time allowed for islanding detection do not take into account the various uncertainties that may arise in a microgrid. In this paper, a new innovative fuzzy based controller is proposed to determine the islanding mode in the event of system uncertainty. The performance of this controller is based on the type-2 fuzzy logic. In general, the capabilities of this method are summarized as follows:

- They do not mis operate in complex operations of the power system.
- They can discriminate the island mode from other network events in a short time.

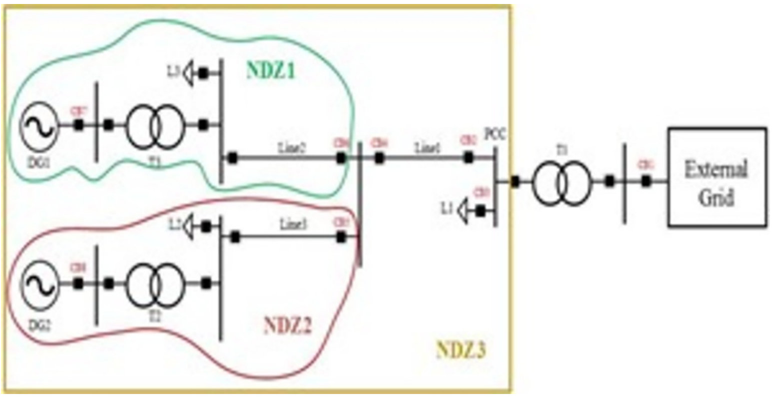

Fig 1. The system under study [40].

https://doi.org/10.1371/journal.pone.0257830.g001

- Uncertainties in the power system (uncertainty in load, system parameters, measuring devices, power generation in DGs) have little effect on the performance of the controller.

The organization of the paper is as follows. Section 2, describes the under-study power system. Uncertainty modeling in microgrids is given in Section 3. Section 4 of the paper introduces the proposed method to identify the islanding mode. Simulation results are provided in Section 5 and finally, conclusion is done in Section 6.

2. The system under study

The single line diagram of the under study microgrid is presented in Fig 1. The system has four buses, two power sources including a wind turbine on bus 1 and a photovoltaic panel installed on bus 2. Other specifications of the studied network are listed in Table 1.

The line between buses 1 and 3 and the line between buses 2 and 3 has a resistance of 1.2 Ω and a reactance of 106 mH. Between buses 3 and 4 the impedance is almost zero.

3. Modeling of uncertainty in microgrids

Studies and research in the field of power systems need to consider uncertainties, the lack of which leads to errors in the results of studies on the network. In this paper, load and DGs production uncertainties are tow main sources of uncertainty in the under-study MG.

3.1. Uncertainty in the network load

In conventional methods for detecting the microgrid islanding, the assumption is that the load is a fixed value, which simplifies implementation but does not correspond to the actual behavior of the load in the power system. Statistical studies proved that consumers' electrical load behavior follows a normal probability density function (PDF). In this distribution, the average value is considered to be the same as the predicted value, and the standard deviation is

Table 1. Specifications of the studied network.

Number of Buses	Number of Line	Rated Frequency (Hz)	Rated Voltage (v)	Load (Kw+jKvar)
4	3	60	400	50+j10

https://doi.org/10.1371/journal.pone.0257830.t001

determined according to the historical information, which is as follows [41].

$$f(P_L) = \frac{1}{\sqrt{2\pi * \sigma_{PL}}} \exp(-\frac{(P_L - \mu_{PL})^2}{2 * \sigma_{PL}^2}) \tag{1}$$

where, P_L, μ_{PL} and σ_{PL} denote the load power (KW), the average value of the load power (KW), and the standard deviation of the load power (KW), respectively.

3.2. Uncertainty in DGs

3.2.1 Wind turbine. Wind speed is random in nature, and to model it, it is necessary to select the probability density function (PDF) or the cumulative probability function (CDF) properly. In this field, many studies and researches have been done and various density probability functions have been tested, such as Weibull, Rayleigh, and Normal probability distribution functions.

In this paper, the Weibull probability distribution function (Eq (2)) is used to model the uncertainty of wind power [42].

$$f_x(v) = \begin{cases} \frac{\beta}{\alpha} \times \left(\frac{v}{\alpha}\right)^{\beta-1} \times \exp\left(-\left(\frac{v}{\alpha}\right)^{\beta}\right) & v \geq 0 \\ 0 & otherwise \end{cases} \tag{2}$$

Where, α (m/s), β, and v (m/s) are scale and shape parameters of the Weibull distribution and wind speed, respectively. These samples are then converted to wind turbine generator output power using the wind speed-power curve (Eq (3)) [42].

$$P_{G,WT}(v) = \begin{cases} 0 & 0 \leq v \leq v_{ci} \ldots or \ldots v \geq v_{co} \\ P_{r.WT} \frac{V - V_{CI}}{V_r - V_{CI}} & v_{ci} < v < v_r \\ P_{r.WT} & v_r < v < v_{co} \end{cases} \tag{3}$$

Where, v_{ci}, v_r and v_{co} are the starting speed v (m/s), nominal speed v (m/s), and cut-off speed v (m/s) of the wind turbine.

3.2.2 PV panels. PV output power is expressed as a function of irradiation as the irradiance power curve, as given in Eq (4) [42]:

$$P_{PV}(R) = \begin{cases} P_{r.PV}(\frac{R^2}{R_{STD} R_C}) & 0 \leq R \leq R_c \\ P_{r.PV}(\frac{R^2}{R_{STD} R_C}) & R_C \leq R \leq R_{STD} \\ P_{r.PV} & R_{STD} \leq R \end{cases} \tag{4}$$

Where, $P_{r.PV}$, R, R_c, and R_{STD} denotes the nominal power of the PV (W/m^2), irradiance, the specific irradiance point (W/m^2) that is usually set to 150 W/m^2, and irradiance in standard conditions (1000 W/m^2).

4. Type-2 fuzzy system

The interval type-2 fuzzy logic system (IT2FLS) consists of two type-1 membership functions, and the distance between these two membership functions indicates uncertainty [7]. As shown in Fig 2, a IT2FLS is described similar to a type-1 fuzzy system using a series of if-then rules, except for the type-2 fuzzy sets use a range (this can be a fuzzy set) in their membership

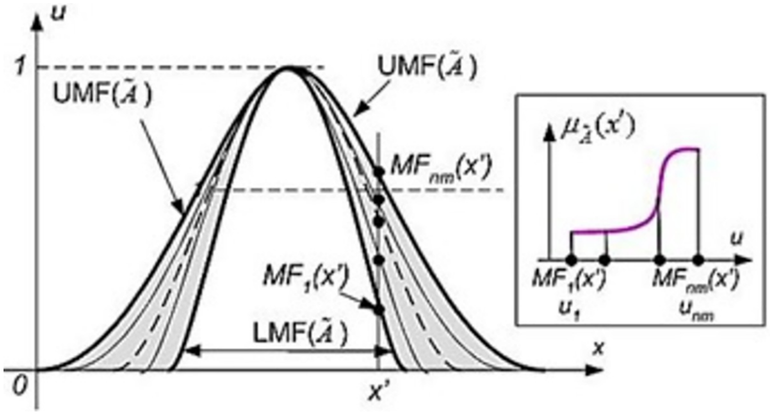

Fig 2. Type-2 membership function.

https://doi.org/10.1371/journal.pone.0257830.g002

functions, instead of employing a number for defining the degree of membership. This range is called the footprint of uncertainties (FOU).

Fig 3 shows the IT2FLS schematic to solve the problem of islanding detection. The IT2FLS includes fuzzification, inference motor, base fuzzy rules, and an output processor.

The type 2 fuzzy blocks are almost the same as the type I fuzzy. But for the IT2FLS a type reducer is needed. The type reducer in the IT2FLS transforms the second type set into a type I fuzzy set. The second type fuzzy set can be defined by Eq (5):

$$\tilde{A} = \{((x,u), \mu_{\tilde{A}}(x,u)) | \forall x \in X, \forall u \in J_x \subseteq [0,1]\} \quad (5)$$

In above equation μ_A (x, u) is the function of the second type fuzzy members in the range of $0 \le \mu_{\tilde{A}}(x,u) \le 1$ and A is defined as Eq (6):

$$\tilde{A} = \int_{x \in X} \int_{u \in J_x} \mu_{\tilde{A}}(x,u)/(x,u) dudx J_x \subseteq [0,1] \quad (6)$$

In Eq (6), J_x is the initial member of x and the μ is variable number in range of 0 and 1. There are two kind of type-2 fuzzy set (General and Interval). For the interval fuzzy set, the value of the second member is always equal to 1. The complexity of general fuzzy design has made it unused in industry and the Interval one is mostly used. In other word, the interval

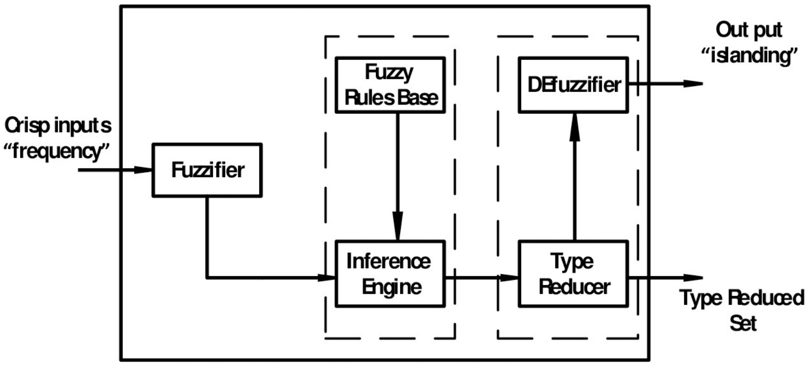

Fig 3. Schematic of the IT2FLS.

https://doi.org/10.1371/journal.pone.0257830.g003

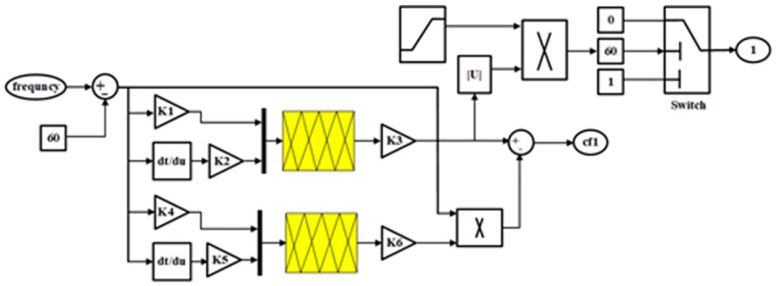

Fig 4. Block diagram of the proposed controller.

https://doi.org/10.1371/journal.pone.0257830.g004

second type fuzzy set is the simplified version of general type which is defined as Eq (7):

$$\mathrm{A} = 1/(x, u)\mathrm{du}\ \mathrm{dx} \quad \subseteq [0, 1] \tag{7}$$

5. The proposed controller

The proposed controller can have a structure such as Fig 4. The proposed controller input is the frequency deviation and its derivative and the controller output is applied to a comparator block to create the appropriate control command. The membership function considered for fuzzy controllers is an interval type-2 fuzzy controller. The main idea for detecting microgrid islanding is to create a range for frequency changes as well as rate of changes over time. It is assumed that the number of changes and the ROCOF for islanding are known, and in the proposed controller, the frequency is first sampled and compared with the reference frequency, then the output of this comparison is amplified and its derivative is obtained. It is then given as two separate inputs to the type-2 fuzzy controller and the controller provides the necessary outputs based on these two inputs.

6. Objective function

The proposed controller can have a structure such as Fig 4. The proposed controller input is the frequency deviation and its derivative and the controller output is applied to a comparator block to create the appropriate control command. The membership function considered for fuzzy controllers is an interval type-2 fuzzy controller. The main idea for detecting microgrid islanding is to create a range for frequency changes as well as rate of changes over time. It is assumed that the number of changes and the ROCOF for islanding are known, and in the proposed controller, the frequency is first sampled and compared with the reference frequency, then the output of this comparison is amplified and its derivative is obtained. It is then given as two separate inputs to the type-2 fuzzy controller and the controller provides the necessary outputs based on these two inputs.

The higher the accuracy of the design, the more efficient the controller. As a result, a meta-heuristic algorithm with a suitable objective function must be used to design the controller. The objective function for the proposed controller designing can be defined as Eq (8):

$$f = \int_{0}^{t_{sim}} (|e|) \times t \tag{8}$$

where, t_{sim} is the simulation time, e is the frequency error value, and t is the time operator. A controller with small values of error and time will perform better.

7. PSO algorithm

The PSO algorithm is a population-based search algorithm and is modeled by imitating the behavior of bird swarms. In this algorithm, the particles in the search space are randomly distributed and the location of the particles in the search space is affected by the experience and knowledge of themselves and their neighbors; thus, the positions of other particles affect how a particle searches the space. The modeling of this social behavior leads to a search process in which particles in successive repetitions tend to successful areas.

The steps for implementing the algorithm are as follows [43].

Step 1: generating the initial population

Generating the initial population is the random determination of the initial positions of the particles with a uniform distribution in the search space.

Step 2: evaluating the objective function

At this step, each particle that represents a solution to the problem must be evaluated. Depending on the problem under consideration, the evaluation method will be different. This is performed by the objective function specific to each problem.

Step 3: determining the best personal particle and the best global particle

After evaluating each particle, the best fitness of each particle ever obtained is stored.

Step 4: updating particles

The new Velocity of the particles is updated using the speed of each particle:

$$V^i_{it+1} = \omega V^i_{it} + c_1 \times rand_1 \times (\mathrm{P}^i_{best} - \mathrm{x}^i_{it}) + c_2 \times rand_2 \times (\mathrm{P}^g_{best} - \mathrm{x}^i_{it}) \tag{9}$$

where P^g_{best} is the best position of the experienced by all the population, w is the weighting factor for the velocity, $rand_1$ and $rand_2$ are two different random numbers, c_1 and c_2 are coefficients of the personal and global best solutions respectively. The new position of the particles is updated by Eq (15):

$$x^i_{it+1} = x^i_{it} + V^i_{it+1} \tag{10}$$

Step 5: Check the termination conditions

Particle position updating continue until the maximum number of iterations is allowed. To terminate the algorithm, a criterion is always set, which can be the maximum number of iterations or the maximum number of iterations without changing the total fitness. The process of implementing the PSO algorithm is shown in Fig 5.

8. Simulation and numerical results

The simulation of the proposed method is performed in MATLAB/Simulink. At t = 6s, islanding is performed using the switch that exists between the microgrid and the upstream power grid. In this microgrid, the solar source includes a PV panel that converts solar power to DC power, and using an IGBT-based inverter, DC power is converted to three-phase AC power. The output power of the inverter is controlled by modulated pulses applied to its gate, and based on the changes in the microgrid frequency, its output power changes, and as a result, its generated power is adjusted to the demand.

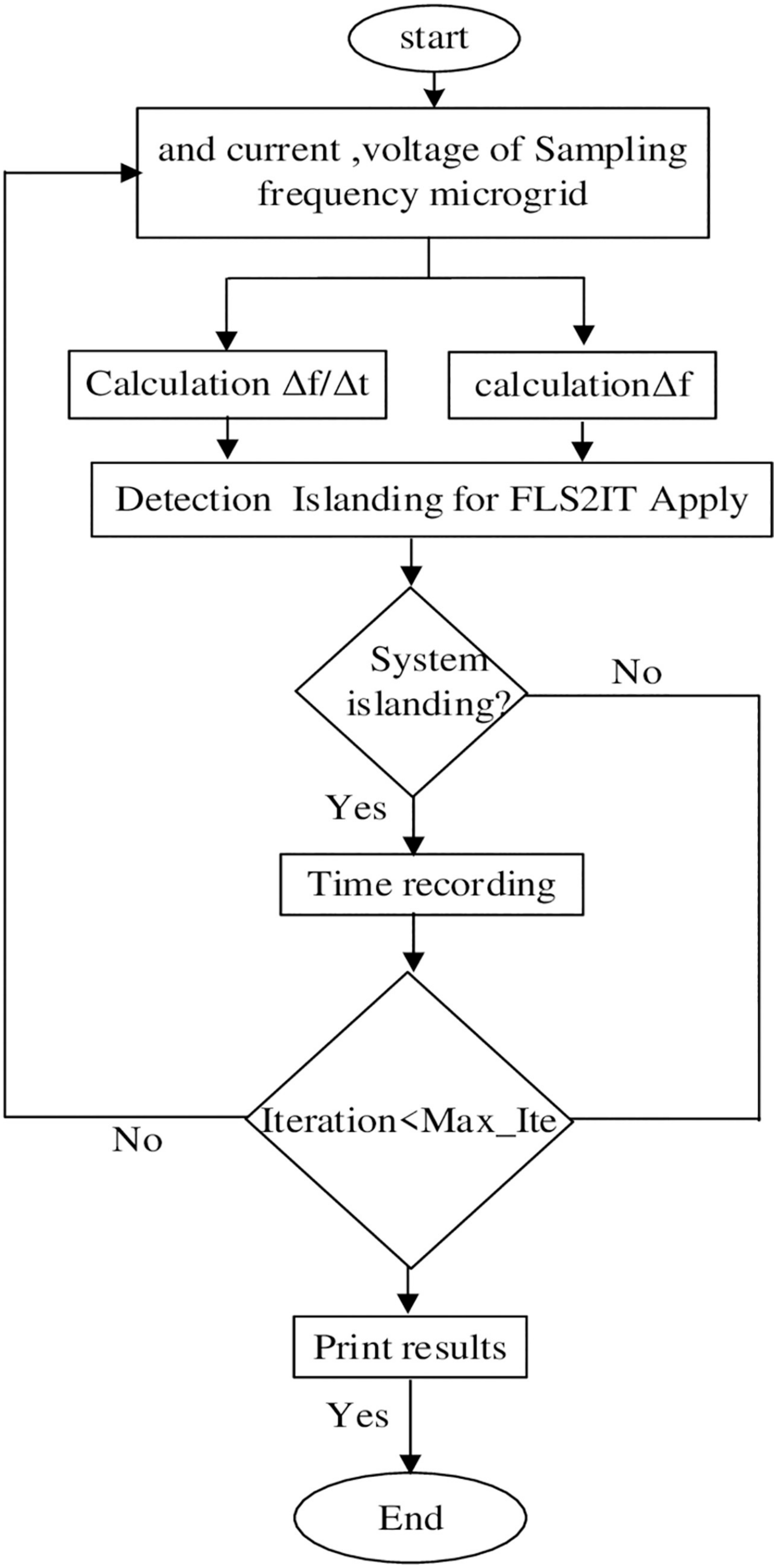

Fig 5. Flowchart of the proposed method.

https://doi.org/10.1371/journal.pone.0257830.g005

Table 2. Fuzzy rules.

Δf/Δt \ Δf	S	M	B
S	N	N	P
M	N	P	P
B	N	P	P

S is small, M is medium and B is big. P is Positive and N is Negative.

https://doi.org/10.1371/journal.pone.0257830.t002

The wind source also includes a wind turbine with the ability to adjust the output power, which changes its output power to match the supply and demand according to the frequency of the system. Table 2 shows the fuzzy rules used to identify islanding.

To consider the uncertainty of wind speed, solar radiation, and load, we change the nominal value to ±25 p.u, with 25 different modes being considered for load demand changes. Furthermore, to investigate the effect of the type of power exchange between the system and the microgrid on frequency changes and islanding detection, we considered the system voltage angles in three different modes: 0 and ±12. In total, 75 different states of uncertainty are considered for each scenario. Four different scenarios are presented to evaluate the proposed method:

Scenario 1) Islanding

Scenario 2) Short circuits in the tie line between the microgrid and the network without islanding

Scenario 3) Interrupting the power generation resources of the microgrid without islanding

Scenario 4) Islanding simultaneous with a sudden increase in the generation power of microgrid resources

8.1. Scenario 1: Islanding

Table 3 shows all possible states, frequency changes during islanding, and whether islanding was identified. Moreover, to investigate the effect of type of power exchange between the system and the microgrid on frequency changes and islanding detection, the system voltage angles are considered in three different modes.

Table 3 shows that in all the cases considered for microgrids and changes in supply and demand, as well as changes in the exchange power between the power system and the distribution network, the islanding detection was well performed. The maximum frequency deviation occurs at 12˚ and the minimum frequency deviation occurs at-12˚. In all cases, when the power output of the wind and solar sources is 1.25p.u. and the system voltage angle is -12˚, the minimum frequency deviation is presented. Also, when the generation is 1.25p.u., the load is 0.75p.u., and the system voltage angle is 12˚, the maximum frequency deviation appears. Therefore, when the MG voltage angle is less than the microgrid voltage angle, it will be more difficult to detect islanding using the proposed method, but in all cases, islanding event is detected. Fig 6 compares several cases of frequency deviation in 10s for the first scenario. In this figure, frequency changes Due to load demand (P_d) changes, Voltage angle, power generation changes are shown in Fig 6(A)–6(C) respectively. It can be observed in Fig 6(A) that the greatest impact on frequency deviation is because of load demand changes and it can be observed in Fig 6(C) that least impact is due to power generation variations. Although increasing the load did not affect the maximum deviation, it did reduce the frequency fluctuations.

Table 3. Results of islanding detection.

		Angle difference 0		Angle difference -12		Angle difference 12	
Generation Variation	Load Variation	Frequency Variation	Islanding Detection	Frequency Variation	Islanding Detection	Frequency Variation	Islanding Detection
1.25	1.25	1.3	Yes	0.94	Yes	1.39	Yes
	1.15	1.32	Yes	1.02	Yes	1.4	Yes
	1	1.33	Yes	1	Yes	1.45	Yes
	0.85	1.41	Yes	1.02	Yes	1.49	Yes
	0.75	1.39	Yes	104	Yes	1.52	Yes
1.15	1.25	1.26	Yes	0.94	Yes	1.37	Yes
	1.15	1.28	Yes	0.95	Yes	1.39	Yes
	1	1.32	Yes	1	Yes	1.4	Yes
	0.85	1.37	Yes	1.06	Yes	1.46	Yes
	0.75	1.39	Yes	1.1	Yes	1.48	Yes
1	1.25	1.28	Yes	0.98	Yes	1.36	Yes
	1.15	1.3	Yes	1	Yes	1.38	Yes
	1	1.31	Yes	1.01	Yes	1.41	Yes
	0.85	1.37	Yes	1.02	Yes	1.43	Yes
	0.75	1.39	Yes	1.07	Yes	1.47	Yes
0.85	1.25	1.27	Yes	0.95	Yes	1.36	Yes
	1.15	1.28	Yes	0.99	Yes	1.37	Yes
	1	1.31	Yes	0.97	Yes	1.39	Yes
	0.85	1.35	Yes	1.03	Yes	1.47	Yes
	0.75	1.38	Yes	1.1	Yes	1.45	Yes
0.75	1.25	1.27	Yes	0.95	Yes	1.36	Yes
	1.15	1.29	Yes	0.96	Yes	1.37	Yes
	1	1.31	Yes	0.97	Yes	1.41	Yes
	0.85	1.32	Yes	1.02	Yes	1.42	Yes
	0.75	1.34	Yes	1.03	Yes	1.46	Yes

https://doi.org/10.1371/journal.pone.0257830.t003

Also, when the network voltage angle is less than that of the microgrid, the frequency deviation is smaller.

To analyze the ability of the presented method, it is compared with three other methods. For this purpose, all 75 modes presented in Table 3 were simulated using type-1 fuzzy logic, neural network, and the neuro-fuzzy methods. Table 3 presents the results of the comparison statistically.

Table 4 shows that the type-2 fuzzy logic is able to detect 100% of the islanding modes, while the other methods have an error percentage, wheretype-1 fuzzy logic method was the most erroneous one. It is also observed that the type-2 fuzzy logic has a higher detection speed than other methods, the neuro-fuzzy method has a better operating time than other methods, and the type-1 fuzzy logic has the lowest speed.

8.2. Scenario 2: Short circuits in the tie line between the microgrid and the network without islanding

In this scenario, three different cases will be considered. In the case 1, the switch that creates the islanding mode at t = 6s is removed, and instead all three phases in tie line that connects the microgrid to the main grid are short circuited. Short circuit occurs at t = 6s and is cleared at t = 7s. In the case 2, islanding does not occur, but a short circuit occurs in the PV bus. In the

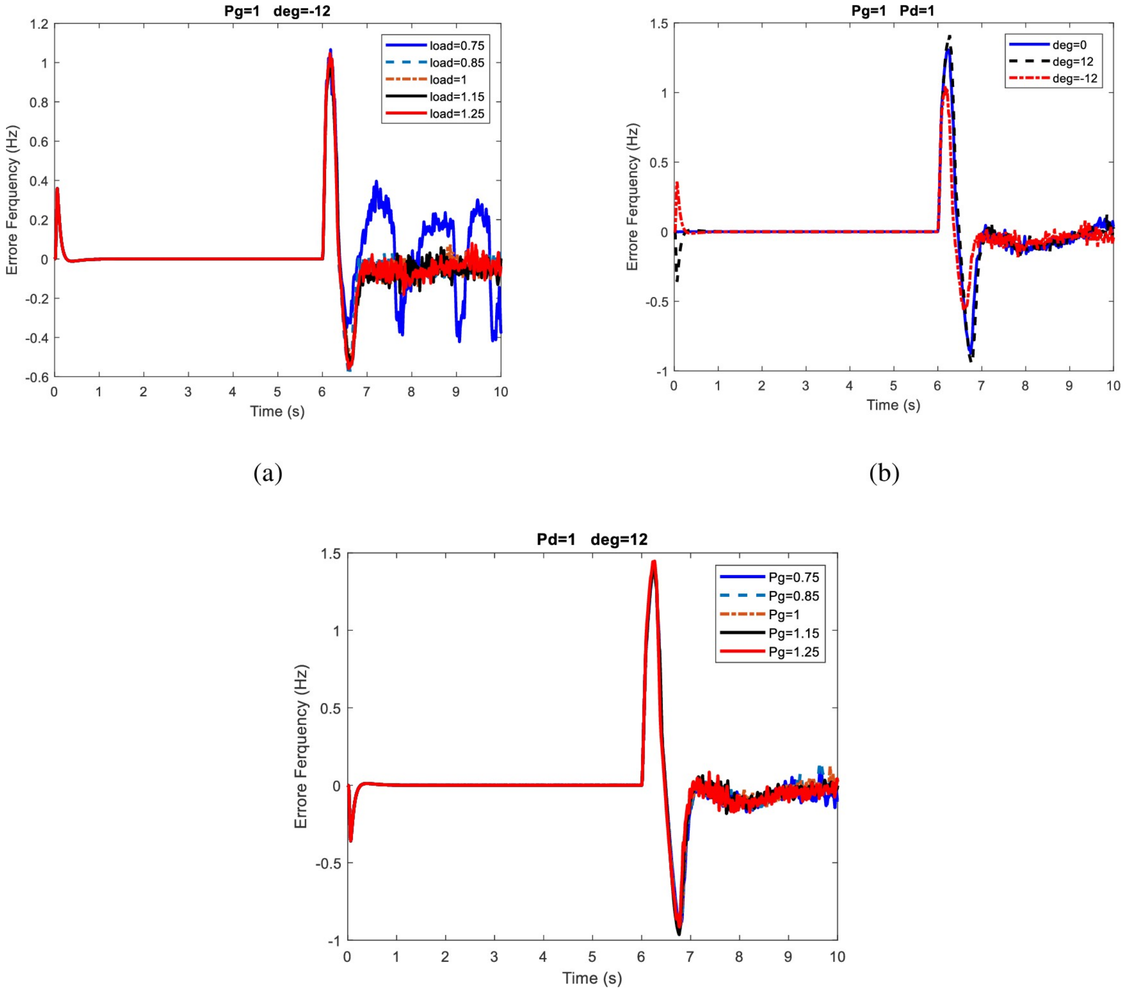

Fig 6. Comparison of frequency deviation in different modes for the first scenario.

https://doi.org/10.1371/journal.pone.0257830.g006

case 3, a short circuit occurs in the WT bus. In these three cases, the islanding mode should not be detected in all 75 cases mentioned above. For this scenario, the proposed method is compared with three other methods, the results are presented in Table 5.

According to the results, the type-2 fuzzy logic is able to correctly identify 100% of the non-islanding modes in all three cases. Also, In the case 2, neural network and neuro-fuzzy methods had equal error percentage, but the neuro-fuzzy method has less error overall. Fig 7 compares several frequency deviations in 10s for the second scenario. In this figure, frequency changes Due to load demand changes, Voltage angle, power generation changes are shown in Fig 7(A)–7(C) respectively. It is observed in Fig 7(A)–7(C) that the effect of load demand changes on frequency deviation is lower than those in Scenario 1, but the effects of changes in the power generation and power exchange with the network had increased, although these changes have not affected the maximum deviation.

Table 4. Comparison of percentage of detection by different methods in scenario 1.

Method	type-2 fuzzy logic system	type-1 fuzzy logic system	neural network	Fuzzy-neural network
Percentage of diagnosis (%)	100	95	96	98
Mean detection time (S)	100	95	97	98

https://doi.org/10.1371/journal.pone.0257830.t004

Table 5. Comparison of percentage of detection by different methods in scenario 2.

Method	type-2 fuzzy logic system	type-1 fuzzy logic system	neural network	Fuzzy-neural network
Case 1	100	95	96	98
Case 2	100	96	97	97
Case 3	100	96	97	98

https://doi.org/10.1371/journal.pone.0257830.t005

8.3. Scenario 3: Interrupting the power generation resources of the microgrid without islanding

In this scenario, three different cases will be considered. In all three cases, the switch that creates the islanding mode at t = 6s is removed and the islanding mode is not created. In the case 1, the PV power source of is disconnected from the microgrid at t = 6s, and all 75 modes mentioned above are examined. In the case of 2, WT source is disconnected from the microgrid at t = 6s and all 75 modes are checked. In the case 3, both WT and PV sources are disconnected from the microgrid at t = 6s and all modes are examined. In this scenario, the proposed method has been compared with three other methods, the results of which have been presented in Table 6.

According to the results, the type-2 fuzzy logic correctly identified 100% of the non-islanding in all three cases, but other methods in this scenario also had an error percentage. In this scenario, the type-1 fuzzy logic method had the highest error in all three cases and the neuro-fuzzy method had the least error. Also, In the case 3, type-1 fuzzy, neural network, and neuro-fuzzy methods had a higher error rate than the cases 1 and 2. Furthermore, in general, in this scenario, the error percentage for these three methods has increased compared to scenarios 1 and 2.

Fig 8 compares several cases of frequency deviation in 10s for the third scenario. In this figure, frequency changes Due to load demand changes, Voltage angle, power generation changes are shown in Fig 8(A)–8(C) respectively. In Fig 8(A), it is observed that the effect of load demand changes on frequency deviation is less than Scenario 1 but higher than Scenario 2. In Fig 8(B) and 8(C), it is recognizable the effects of changes in power generation and power exchange with network have increased compared to previous scenarios.

8.4. Scenario 4: Islanding simultaneous with a sudden increase in the generation power of microgrid resources

In this scenario, three different cases are examined, and in all three cases, the switch that creates the islanding mode at t = 6s, establishes the islanding mode. In the case 1, the PV source experiences a sudden change of 70% at t = 6s and all 75 states mentioned above are investigated. In the case 2, the WT sources experiences a sudden change of 70% at t = 6s and all 75 states are investigated. In the case 3, both WT and PV sources face a sudden change of 70% at t = 6s and all 75 states are examined. In this scenario, the proposed method has been compared with three other methods, the results are presented in Table 7.

The results accumulated in Table 7 indicate that the type-2 fuzzy logic in all three cases correctly identified 100% of the non-islanding but other methods in this scenario also had an error percentage. In this scenario, the type-1 fuzzy logic method had the highest error in all three cases and the neural-fuzzy method had the least error. Also, In the case 3, type-1 fuzzy, neural network, and neuro-fuzzy method had a higher error percentage than cases 1 and 2. Also, in general, in this scenario, the error percentage in these three methods has increased compared to first and second scenarios.

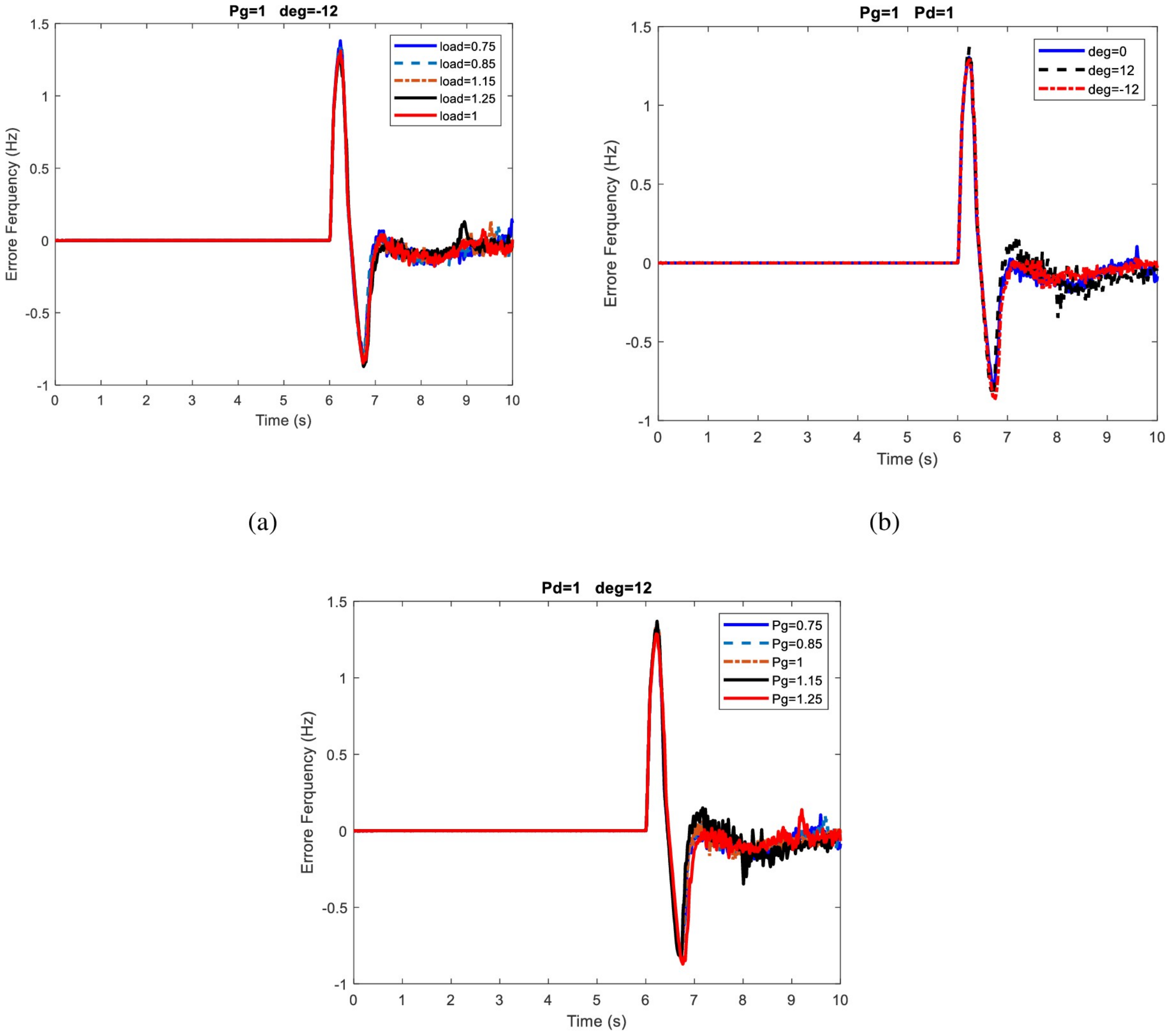

(a) (b)

(c)

Fig 7. Comparison of frequency deviations in different modes for the second scenario.

https://doi.org/10.1371/journal.pone.0257830.g007

Fig 9 compares several cases of frequency deviation in 10s for the fourth scenario. In this figure, frequency changes Due to load demand changes, Voltage angle, power generation changes are shown in Fig 9(A)–9(C) respectively. The effect of load demand changes (Fig 9 (A)), power generation changes (Fig 9(B)), and power exchange changes (Fig 9(A)) on frequency deviation has been greatly increased. It can even be seen that changes in power generation and power exchange changes affect the maximum frequency deviation.

Table 6. Comparison of percentage of detection by different methods in scenario 3.

Method	type-2 fuzzy logic system	type-1 fuzzy logic system	neural network	Fuzzy-neural network
**Case 1**	100	94	95	98
**Case 2**	100	95	97	98
**Case 3**	100	93	94	97

https://doi.org/10.1371/journal.pone.0257830.t006

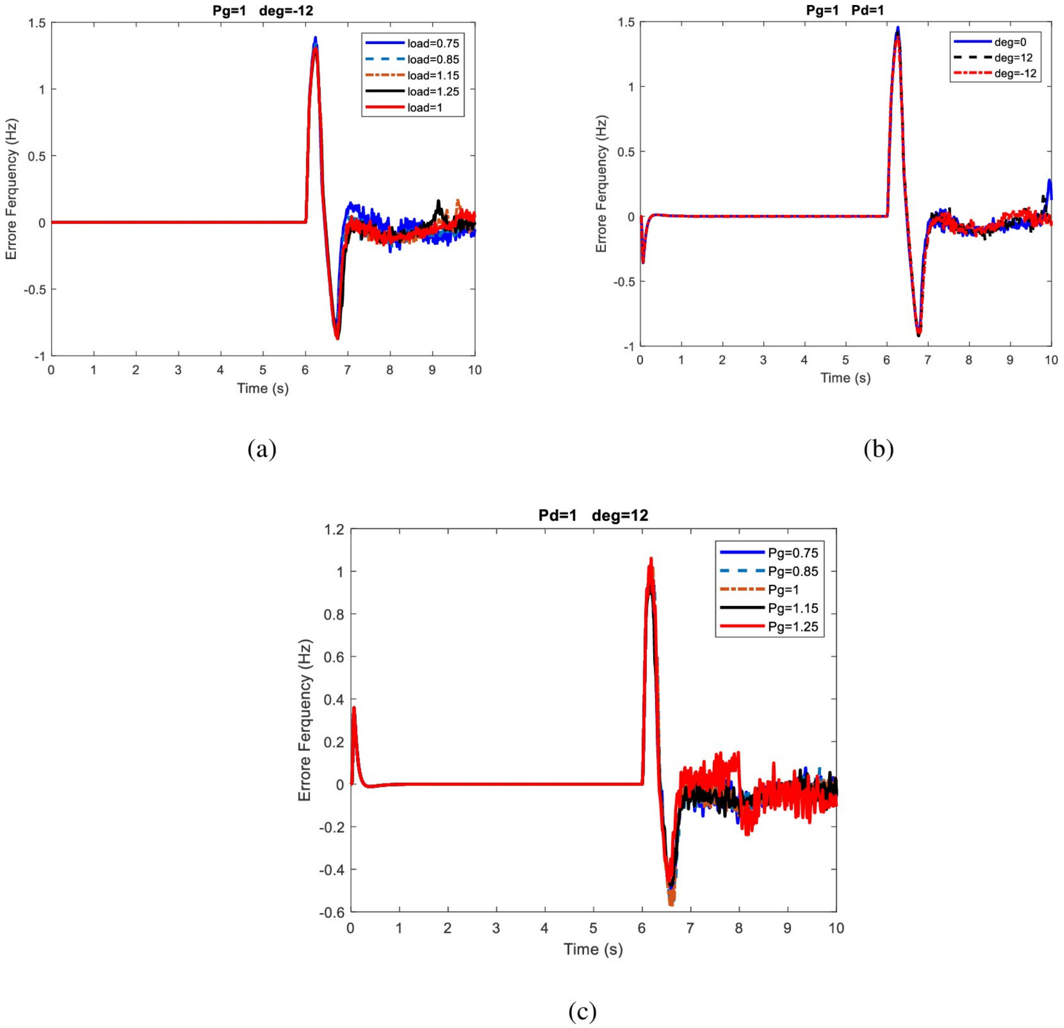

Fig 8. Comparison of frequency deviation in different modes for the third scenario.

https://doi.org/10.1371/journal.pone.0257830.g008

9. Conclusion

This paper presents a novel method for detecting islanding using a combination of type-2 fuzzy logic and PSO optimization algorithm based on microgrid frequency changes in situations where the production of wind and solar resources as well as load consumption are uncertain. The proposed method was simulated on a sample system in MATLAB software and different scenarios were considered. In each scenario, 75 different modes of changes in power generation, power consumption, and power exchange between the microgrid and the main network were presented. In all cases, the proposed method was able to identify islanding. Also,

Table 7. Comparison of percentage of detection by different methods in scenario 3.

Method	type-2 fuzzy logic system	type-1 fuzzy logic system	neural network	Fuzzy-neural network
**Case 1**	100	95	96	98
**Case 2**	100	95	96	98
**Case 3**	100	96	97	98

https://doi.org/10.1371/journal.pone.0257830.t007

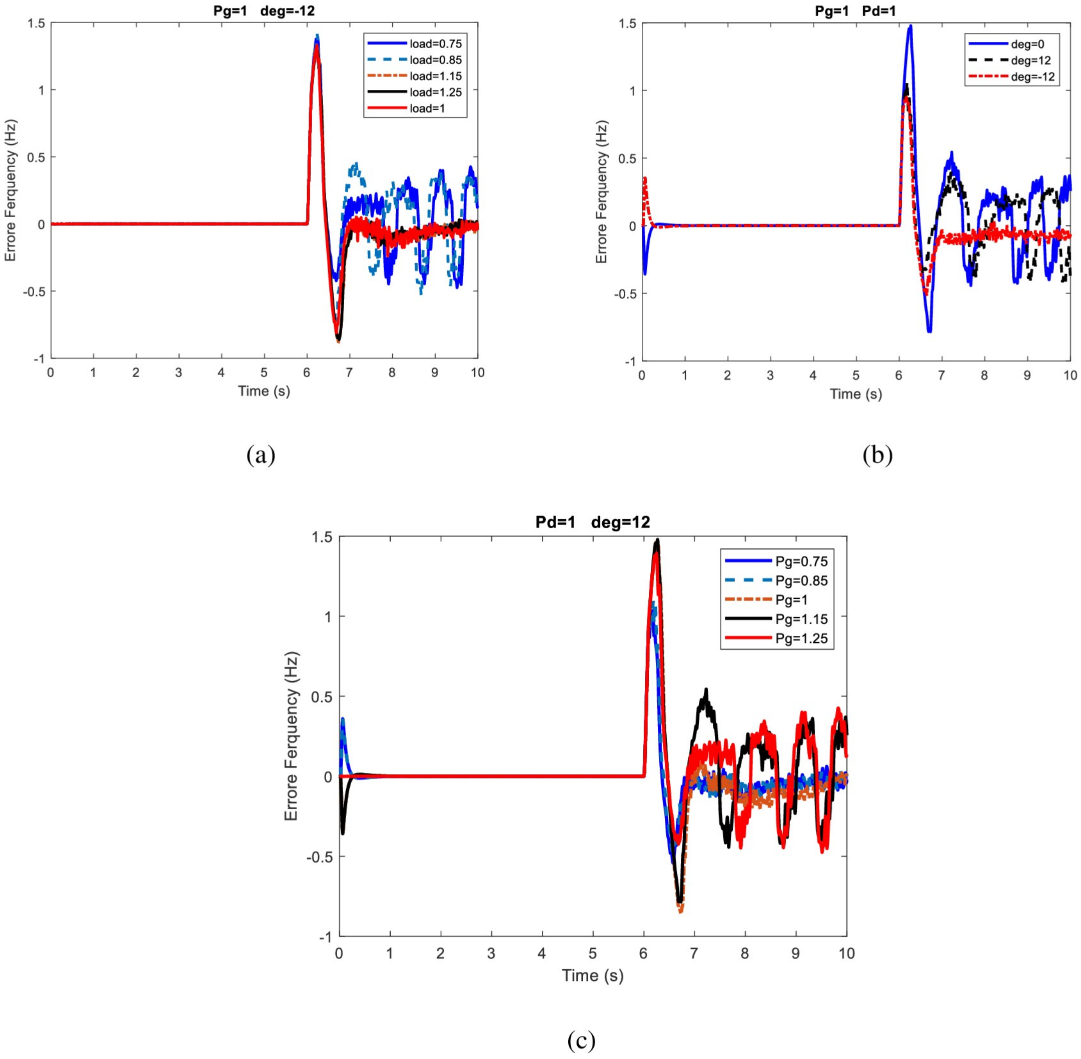

Fig 9. Comparison of frequency deviation in different cases for the fourth scenario.

https://doi.org/10.1371/journal.pone.0257830.g009

the type-2 fuzzy system is superior to the type-1 fuzzy logic in supporting noise conditions, changes in the environment, and the presence of uncertainty because its membership degree is a fuzzy set itself. Eventually, the proposed method did not mis operate for any case or scenario, while other methods such as type-1 fuzzy logic, neural network, and neuro-fuzzy methods always had error percentage in identifying whether or not an islanding has occurred.

Author Contributions

Conceptualization: Mehrdad Ahmadi Kamarposhti.

Data curation: Mehrdad Ahmadi Kamarposhti.

Formal analysis: Mehrdad Ahmadi Kamarposhti.

Methodology: Taghi Hosseinzadeh Khonakdari, Mehrdad Ahmadi Kamarposhti.

Project administration: Mehrdad Ahmadi Kamarposhti.

Resources: Mehrdad Ahmadi Kamarposhti.

Software: Taghi Hosseinzadeh Khonakdari, Mehrdad Ahmadi Kamarposhti.

Supervision: Mehrdad Ahmadi Kamarposhti.

Validation: Taghi Hosseinzadeh Khonakdari, Mehrdad Ahmadi Kamarposhti.

Visualization: Taghi Hosseinzadeh Khonakdari, Mehrdad Ahmadi Kamarposhti.

Writing – original draft: Taghi Hosseinzadeh Khonakdari, Mehrdad Ahmadi Kamarposhti.

Writing – review & editing: Mehrdad Ahmadi Kamarposhti.

References

1. Poluektov C., Anton, et al. "Sensitivity analysis of a PLC-based DSSS anti-islanding system in power distribution grids." International Journal of Electrical Power & Energy Systems 113 (2019): 739–747.
2. Anudeep, Bhatraj, Aman Verma, and Paresh Kumar Nayak. "Comparative Assessment of Passive Islanding Detection Techniques for Distributed Generations." Proceedings of the Third International Conference on Microelectronics, Computing and Communication Systems. Springer, Singapore, 2019.
3. Ku Ahmad Ku Nurul Edhura, Selvaraj J, Rahim NA. "A review of the islanding detection methods in grid connected PV inverters," Renew Sustain Energy Rev, Vol. 21, pp. 756–766, 2013.
4. Bakhshi-Jafarabadi Reza, Ghazi Reza, and Sadeh Javad. "Power Quality Assessment of Voltage Positive Feedback Based Islanding Detection Algorithm." Journal of Modern Power Systems and Clean Energy (2020).
5. Kermany S.D., Joorabian M., Deilami S., Masoum M.A.S., "Hybrid Islanding Detection in Microgrid with Multiple Connection Points to Smart Grids using Fuzzy-Neural Network," IEEE Trans on Power Systems, Vol. 32 Issue: 4, pp. 2640–2651, 2017.
6. Reddy Ch Rami, Reddy K. Harinadha, and Siva Reddy K. Venkata. "Recognition of islanding data for multiple distributed generation systems with ROCOF shore up analysis." Smart Intelligent Computing and Applications. Springer, Singapore, 2019. 547–558.
7. Chandio Asad Ali, et al. "A new islanding detection technique based on rate of change of reactive power and radial basis function neural network for distributed generation." Journal of Intelligent & Fuzzy Systems 37.2 (2019): 2169–2179.
8. Nale Ruchita, Biswal Monalisa, and Kishor Nand. "A Transient Component Based Approach for islanding detection in distributed generation." IEEE Transactions on Sustainable Energy (2018). https://doi.org/10.1109/TSTE.2018.2789685 PMID: 30405893
9. Raza S., Mokhlis H., Arof H., Laghari J., and Wang L., "Application of signal processing techniques for islanding detection of distributed generation in distribution network: A review," Energy Conversion and Management, Vol. 96, pp. 613–624, 2015.
10. Zhao, Yucan, et al. "A delay triggered reactive power perturbation method for islanding detection of grid-connected PV power generation systems." 2019 IEEE Sustainable Power and Energy Conference (iSPEC). IEEE, 2019.
11. Hashemi F.; Mohammadi M.; Kargarian A., "Islanding detection method for microgrid based on extracted features from differential transient rate of change of frequency," IET Generation,Transmission & Distribution, Vol. 11, pp. 891–904,2017.
12. Gupta Pankaj, Bhatia R. S., and Jain D. K. "Active Islanding Detection Technique for Distributed Generation." INAE Letters 3.4 (2018): 243–250.
13. Zeineldin HH, Kennedy S, "Sandia frequency-shift parameter selection to eliminate non detection zones,"Renew Power Gener IET, Vol. 5, pp. 175–83, 2011.
14. Stevens J, Bonn R, Ginn J, Gonzalez S, Kern G, "Development and testing of an approach to antiislanding in utility-interconnected photovoltaic systems," Sandia National Laboratories, report SAND2010. Albuquerque, NM: Sandia National Laboratories; 2010.
15. Jalakas, Tanel, Indrek Roasto, and Mahdiyyeh Najafzadeh. "Combined Active Frequency Drift Islanding Detection Method for NZEB Energy Router." 2019 IEEE 60th International Scientific Conference on Power and Electrical Engineering of Riga Technical University (RTUCON). IEEE, 2019.
16. Hernandez-Gonzalez G, Iravani R, "Current injection for active islanding detection of electronically-interfaced distributed resources," IEEE Trans Power Deliv, Vol. 5, pp. 1698–705, 2016.
17. Muda Harikrishna, and Jena Premalata. "Phase angle-based PC technique for islanding detection of distributed generations." IET Renewable Power Generation 12.6 (2018): 735–746.

18. Gupta Nikita, and Garg Rachana. "Algorithm for islanding detection in photovoltaic generator network connected to low-voltage grid." IET Generation, Transmission & Distribution 12.10 (2018): 2280–2287.
19. Reigosa D, Briz F, Blanco C, Garcia P, Manuel Guerrero J, "Active islanding detection for multiple parallel-connected inverter-based distributed generators using high-frequency signal injection," IEEE Trans Power Electron, Vol. 29, pp. 1192–9, 2014.
20. Dolatabadi Soheil, Tohidi Sajjad, and Ghasemzadeh Saeed. "A New Active Method for Islanding Detection Based on Traveling Wave Theory." Iranian Journal of Electrical and Electronic Engineering 14.4 (2018): 382.
21. Chiang W-J, Jou H-L, Wu J-C, "Active islanding detection method for inverter based distribution generation power system," Int J Electr Power Energy Syst, Vol. 42, pp. 158–66, 2012.
22. Ma, Cong, et al. "Power Perturbation Method for the Voltage Ride Through Operation and Islanding Detection of Grid-Tied Renewable Energy Generation Systems." 2019 IEEE Sustainable Power and Energy Conference (iSPEC). IEEE, 2019.
23. Pourbabak H, Kazemi A, "A new technique for islanding detection using voltage phase angle of inverter-based DGs," Int J Electr Power Energy Syst, Vol. 57, pp. 198–205, 2014.
24. Indu Rani B, Srikanth M, Saravana Ilango G, Nagamani C, "An active islanding detection technique for current controlled inverter," Renew Energy, Vol. 51, pp. 189–96, 2013.
25. Sun Q., Guerrero J. M., Jing T., Vasquez J. C., Yang R., "An Islanding Detection Method by Using Frequency Positive Feedback Based on FLL for Single-Phase Microgrid," IEEE Transactions on Smart Grid, Vol. 8, pp. 1821–1830, 2017.
26. Mlakić Dragan, Baghaee Hamid Reza, and Nikolovski Srete. "Gibbs Phenomenon-based Hybrid Islanding Detection Strategy for VSC-based Microgrids using Frequency Shift, THDU and RMSU." IEEE Transactions on Smart Grid (2018).
27. Seyedi Masoumeh, et al. "A hybrid islanding detection technique for inverter-based distributed generator units." International Transactions on Electrical Energy Systems (2019).
28. Laghari JA, Mokhlis H, Bakar AHA, Karimi M. "A new islanding detection technique for multiple mini hydro based on rate of change of reactive power and load connecting strategy," *Energy Convers Manag*, Vol. 76, pp. 215–224, 2013.
29. Ghzaiel W., Ghorbal M.J.B., Belkhodja I.S., Guerrero J.M., "Grid impedance estimation based hybrid islanding detection method for AC microgrids" *Elsevier Journal*, *International Association for Mathematics and Computers in Simulation (IMACS)*, Vol. 131, pp. 142–156, 2017.
30. Narayanan K., Shahbaz A S, Manoj F, "Hybrid islanding detection method and priority-based load shedding for distribution networks in the presence of DG units," *IET Journals & Magazines*, *Generation*, *Transmission & Distribution*, Vol. 11, Issue 3, pp. 586–595, 2017.
31. A. Azizi, S.M.T. Bathaee, "Hybrid islanding detection method by virtual synchronous generator in microgrids with flexible DG," in Proc. IEEE Conf. Electrical Engineering (ICEE), Tehran, pp. 757–762, 2014.
32. Heidari M, Seifossadat G, Razaz M, "Application of decision tree and discrete wavelet transform for an optimized intelligent-based islanding detection method in distributed systems with distributed generations," Renew Sustain Energy Rev, Vol. 27, pp. 525–32, 2013.
33. Baghaee Hamid Reza, et al. "Support Vector Machine-based Islanding and Grid Fault Detection in Active Distribution Networks." IEEE Journal of Emerging and Selected Topics in Power Electronics (2019).
34. Pinto Smitha Joyce Panda Gayadhar,"Wavelet technique based islanding detection and improved repetitive current control for reliable operation of grid-connected PV systems," Int J Electr Power Energy Syst, Vol. 67, pp. 30–51, 2015.
35. Samantaray SR, El-Arroudi K, Joos G, Kamwa I, "A fuzzy rule-based approach for islanding detection in distributed generation," IEEE Trans Power Deliv, Vol. 25, pp. 1427–33, 2010.
36. Li Yang, et al. "Islanding fault detection based on data-driven approach with active developed reactive power variation." Neurocomputing 337 (2019): 97–109.
37. Kim Jisoo, et al. "An Islanding Detection Method for Multi-RES Systems using the Graph Search Method." IEEE Transactions on Sustainable Energy (2020).
38. Bakhshi-Jafarabadi Reza, Sadeh Javad, and Popov Marjan. "Maximum Power Point Tracking Injection Method for Islanding Detection of Grid-Connected Photovoltaic Systems in Microgrid." IEEE Transactions on Power Delivery (2020).
39. Prakash Satya, Purwar Shubhi, and Mohanty Soumya R. "Adaptive Detection of Islanding and Power Quality Disturbances in a Grid-Integrated Photovoltaic System." Arabian Journal for Science and Engineering (2020): 1–14.

40. Khamis Aziah, et al. "Faster detection of microgrid islanding events using an adaptive ensemble classifier." IEEE Transactions on Smart Grid 9.3 (2016): 1889–1899.

41. Nikmehr Nima, and Ravadanegh Sajad Najafi. "Heuristic probabilistic power flow algorithm for microgrids operation and planning." IET generation, transmission & distribution 9.11 (2015): 985–995.

42. Bukhari Syed Basit Ali, et al. "An interval type-2 fuzzy logic based strategy for microgrid protection." International Journal of Electrical Power & Energy Systems 98 (2018): 209–218.

43. Nikmehr Nima, and Najafi-Ravadanegh Sajad. "Optimal operation of distributed generations in microgrids under uncertainties in load and renewable power generation using heuristic algorithm." IET Renewable Power Generation 9.8 (2015): 982–990. https://doi.org/10.1049/iet-rpg.2014.0357
